# Surface Modification of Staple Carbon Fiber by Dopamine to Reinforce Natural Latex Composite

**DOI:** 10.3390/polym12040988

**Published:** 2020-04-24

**Authors:** Xiaolong Tian, Shuang Han, Qianxiao Zhuang, Huiguang Bian, Shaoming Li, Changquan Zhang, Chuansheng Wang, Wenwen Han

**Affiliations:** 1 College of Electromechanical Engineering, Qingdao University of Science and Technology, Qingdao 266061, Shandong Province, China; 15165268516@163.com (X.T.); hanshuang258@163.com (S.H.); zqx_112@163.com (Q.Z.); bhg@qust.edu.cn (H.B.); jdwz@qust.edu.cn (S.L.); qw18561929710@163.com (C.Z.); 2 Academic Division of Engineering, Qingdao University of Science & Technology, Qingdao 266061, Shandong Province, China; 3 National Engineering Laboratory for Advanced Tire Equipment and Key Materials, Qingdao University of Science and Technology, Qingdao 266061, Shandong Province, China

**Keywords:** surface modification of staple carbon fiber, natural rubber latex, reinforcement mechanism, dopamine, rubber composite

## Abstract

Carbon fiber significantly enhances the mechanical, thermal and electrical properties of rubber composites, which are widely used in aerospace, military, national defense and other cutting-edge fields. The preparation of a high-performance carbon fiber rubber composite has been a research hotspot, because the surface of carbon fiber is smooth, reactive inert and has a poor adhesion with rubber. In this paper, a high-performance rubber composite is prepared by mixing dopamine-modified staple carbon fiber with natural latex, and the mechanisms of modified carbon fiber-reinforced natural latex composite are explored. The experimental results show that the surface-modified staple carbon fiber forms uniform and widely covered polydopamine coatings, which significantly improve the interface adhesion between the carbon fiber and the rubber matrix. Meanwhile, when the concentration of dopamine is 1.5 g/L and the staple carbon fiber is modified for 6h, the carbon fiber rubber composite shows excellent conductivity, thermal conductivity, and dynamic mechanical properties, and its tensile strength is 10.6% higher than that of the unmodified sample.

## 1. Introduction

Carbon fiber (CF) is formed by the thermal conversion of organic fibers, and has a high strength, high modulus, high thermal conductivity, chemical corrosion resistance and other excellent properties [1,2]. At present, CF-reinforced rubber composites (CFRC) have been widely used in aerospace, military, national defense, automobile and other fields due to their excellent properties [3,4,5,6]. A good interface between the CF and the rubber matrix is significantly difficult to obtain, and this plays a crucial role in preparing high-performance CFRC. This has also resulted in extensive research by scholars at home and abroad.

The surface modification of CF has become the main method to enhance the interfacial adhesion, which can be divided into physical modification and chemical modification [7,8,9,10]. The main function is to improve the surface roughness of the CF and increase the meshing effect between the CF and the matrix, on the other hand, to introduce active groups on the surface of carbon fiber to enhance the interaction between the CF and the rubber matrix [11,12,13,14]. As a new surface modification material, the application of dopamine is gradually expanding [15,16]. Kim et al. [17] found that, under the condition of high oxygen concentration, dopamine can be evenly deposited in a short time, and a smooth layer of dopamine can be obtained. Win et al. [18] proved that the addition of oxidants can greatly shorten the self-polymerization time of dopamine. Du et al. [19] used ultraviolet radiation to control the self-polymerization of dopamine.

**Figure 1:**
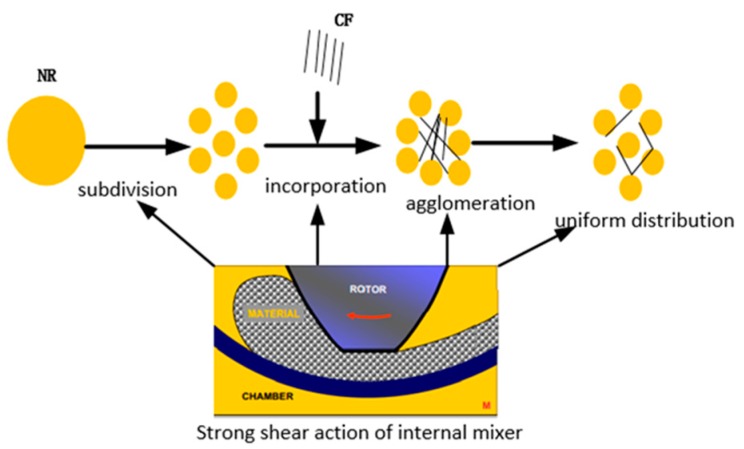
The processing of carbon fiber-reinforced rubber composites (CFRCs).

The processing method of CFRC is also an important factor affecting the interfacial adhesion. In the traditional processing, the natural rubber (NR) subdivision, CF incorporation, agglomeration of CF and uniform distribution of CF successively occurs, which mainly depends on the strong shear force provided by the internal mixer in order to realize the uniform dispersion of CF in the rubber matrix, as shown in Figure 1. CF is easy to agglomerate and is broken in the traditional processing method, which decreases the properties of CFRC. To prepare high-performance CFRC, natural rubber latex (NRL), which has a good film-forming performance and can evenly cover CF to form a film, replaces NR to reinforce the interfacial adhesion between the CF and the rubber matrix.

The present work investigates the difference in the properties of CFRC prepared by NRL or NR and verifies the advantages of wet mixing NRL and CF to prepare NRL/CF composites. Consequently, the mechanisms of dopamine surface-modified carbon fiber-reinforced natural rubber latex composites are systematically studied. Furthermore, the effects of dopamine concentration and modification time on the properties of composites are studied.

## 2. Materials and Methods

### 2.1. Experimental Method

Table 1 shows the experimental scheme, exploring the influence of dopamine surface-modified CF on the properties of CFRC.

### 2.2. Materials

Table 2 shows the composites’ formulations and suppliers.

### 2.3. Adhesion Mechanism of CF and NRL

**Figure 2:**
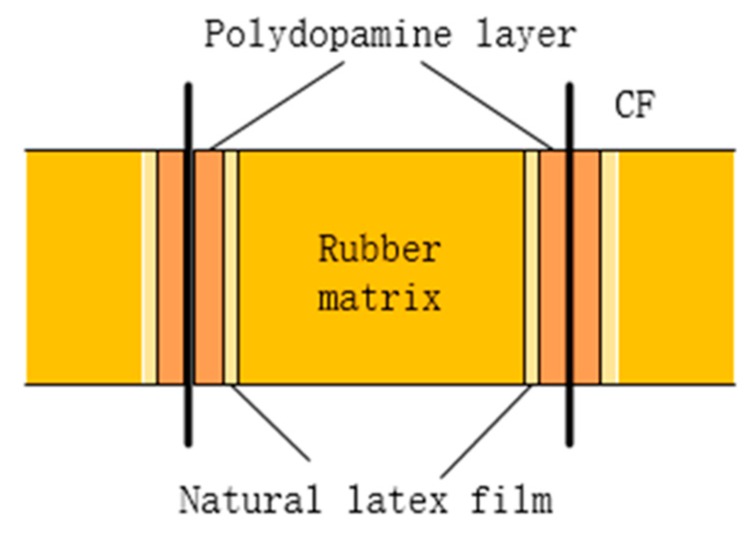
The adhesion interface between the CF and rubber matrix.

The interface behavior between the CF and the rubber matrix is a key factor affecting the performance of composite materials. A polydopamine layer was grown on the surface of CF, after being modified by dopamine, which was covered by a latex film formed by natural latex, and then bonded with the rubber matrix to produce good interfacial interaction. Figure 2 shows the adhesion interface between the CF and the rubber matrix.

**Figure 3:**
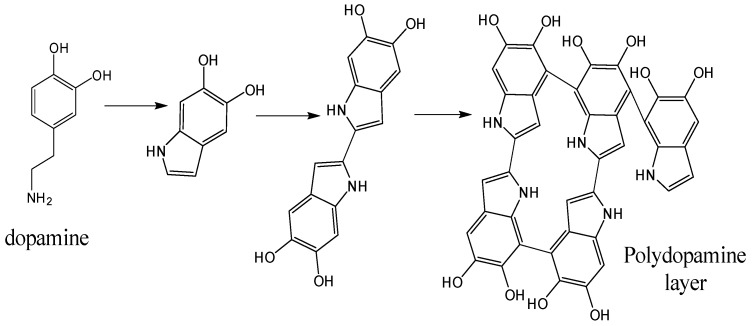
The formation process of the polydopamine layer.

Dopamine surface-modified carbon fiber can enhance the bonding effect between the CF and the rubber matrix. Dopamine can be oxidized and self-polymerized in alkaline aqueous solution, forming a polydopamine layer and a covalent bond with double bond groups in rubber molecules, while binding with CF through intermolecular interaction, which involves a Van der Waals interaction, hydrogen bond, and other non-covalent interactions, thus forming a good interface effect. The formation process of the polydopamine layer is shown in Figure 3.

### 2.4. Preparation of Composite Materials

#### 2.4.1. Pretreatment of the Material

**(1) Impregnate CF with NRL:**

Use ultrasound (VCY-1500, Shanghai Yanyong Chaosheng Equipment Co., Ltd., Shanghai, China; experimental parameters: ultrasonic power, 1000 W; ultrasonic time, 5 min) to vibrate the NRL to destroy the protein layer and phospholipid layer of the latex particles. Clean the CF with deionized water and add it to the treated NRL. Use the high-speed disperser (T 25 easy clean digital, German IKA company, Staufen im Breisgau, Germany; rotation rate: 400 rpm) to mix the composite for 10 min, and then pour it into a large tray and tile it for drying in order to prepare the NRL/CF masterbatch.

**(2) Modify CF with different concentrations of dopamine:**

Prepare dopamine solutions with concentrations of 0.5, 1, 1.5 and 2 g/L. Add the Tris (Trihydroxymethyl aminomethane, purchased from Shanghai Aladdin Biochemical Technology Co., Ltd., Shanghai, China) to the dopamine solution and adjust the pH to around 8.5. Clean the CF with deionized water, and add it into different concentrations of dopamine, heating for 2 h in a water bath. Then, clean the modified CF by deionized water again, in order to mix it with the NFL treated by ultrasound.

**(3) Modify CF with different processing times of dopamine:**

Clean the CF with deionized water and add the CF into four dopamine solutions with a concentration of 1.5 g/L, heating for 2 h in a water bath. Then, clean the modified CF by deionized water again, in order to mix it with the NFL treated by ultrasound.

#### 2.4.2. Mixing Processing

**(1) NR/CF composite:**

CF, NR and other fillers were incorporated into the mixer for mixing. The mixing process was as follows: the rotating speed was 60 rpm, the filling coefficient of the mixer was 0.7, and the cooling water temperature was 60 °C. 

Sulfur and accelerant were incorporated into the open mill to prepare the compound of NR/CF composites.

**(2) NRL/CF composite:**

(a) The mixture of modified CF and NRL treated by ultrasound was mixed by the high-speed disperser for 10 min, and then drying to prepare NRL/CF masterbatch;

(b) The masterbatch and other fillers were incorporated into the mixer according to the mixing process of the compound of NRL/CF composites. 

Sulfur and accelerant were incorporated into the open mill to prepare the compound of NRL/CF composites.

#### 2.4.3. Curing Process

The rubber compounds were cured at 150 °C at a pressure of 10 MPa for an optimum cure time (t90) + 3 min.

### 2.5. Characterization

**Curing Characterization.** The curing characterization of rubber compounds was evaluated using a moving-die rheometer (M-2000-AN) from GOTECH TESTING MACHINES CO., Ltd. The specimens were tested according to ISO 6502-2: 2018. The Mooney viscosity values of the rubber compounds were evaluated using a Mooney viscometer (UM-2050, GOTECH TESTING MACHINES CO., Ltd., Taichung, Taiwan) according to ISO 289-2: 2016.

**Physical and Mechanical Properties.** The hardness of the rubber vulcanizate was evaluated using Shore Hardmeter (LX-A, Shanghai Liuling Instrument Factory, Shanghai, China) according to ISO 7619-2: 2004, three points were measured for each sample and the final result produced the median [6]. The tensile and tear properties of the vulcanized rubber were tested using a universal testing machine (TS 2005 b, GOTECH TESTING MACHINES CO., Ltd., Taichung, Taiwan) at a drawing rate of 500 mm/min according to the standards ISO 37: 2005 and ISO 34-1: 2004, respectively [20]. Five specimens were tested for each sample type and the final result produced the median. The abrasion of the rubber vulcanizates were evaluated using a DIN wear machine (GT-2012-D, GOTECH TESTING MACHINES CO., Ltd., Taichung, Taiwan) according to GB/T 1689-1998, three specimens were tested and the final result produced the median.

**Dynamic Mechanical Thermal Analysis.** The viscoelastic mechanical properties of the vulcanizates were evaluated using a dynamic thermomechanical analyzer (EPLEXOR-150N, Gabo Qualimeter Testanlagen GmbH, Ahlden, Germany), in which the test temperature range was −65 to 65 °C, the heating rate was 2 K/min, the vibration frequency was 10 Hz, the static strain was 5%, the static force was 70 N, the dynamic strain was 0.25%, and the dynamic stress was 60 N. 

**Morphology Analysis.** The cross-section of the sample after tensile fracture, which adhered to the conductive adhesive and was fixed on the sample table for gold spraying, was observed under a scanning electron microscope (JSM-7500F, Japan Electronics Corporation).

**Payne effect.** The Payne effect of the composites was tested by the rubber processing analyzer, in which the scanning conditions were set at 60 °C, the scanning frequency was 1 Hz, and the scanning range was 0.28%–40%.

## 3. Results and Discussion

### 3.1. Dopamine Surface-Modified CF on the Microstructure of the Composite

The cohesion interaction between the CF and the rubber matrix can be seen clearly from the electron microscope image, which is important for the evaluation of surface-modified CF by dopamine. 

**Figure 4:**
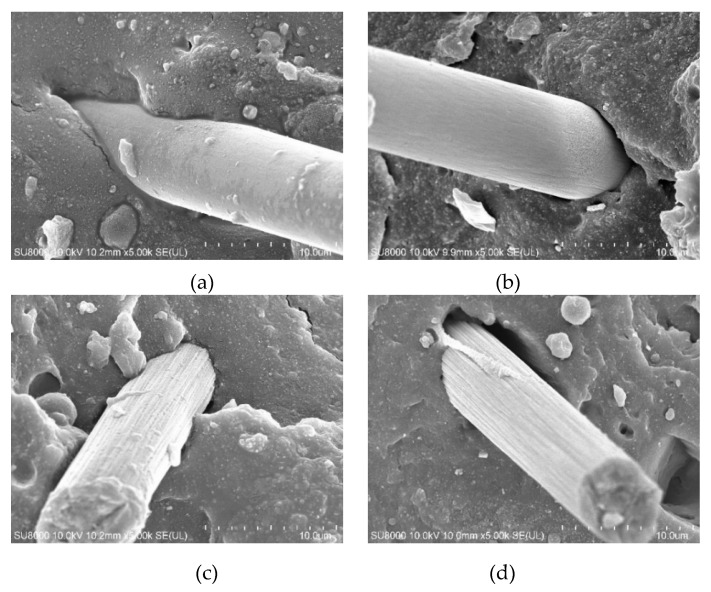
SEM pictures of CF in natural rubber (NR) and natural rubber latex (NRL): (**a**) SEM pictures of NRL/CF; (**b**) SEM pictures of NR/CF; (**c**) SEM pictures of NRL/CF; (**d**) SEM pictures of NR/CF.

The CF is impregnated with NRL to form an adhesive film on its surface, which strengthens the adhesion between the CF and NRL from Figure 4. There is a large gap between the CF and the rubber matrix in the composite of NR/CF, which weakens the interface adhesion between the CF and the rubber matrix.

**Figure 5:**
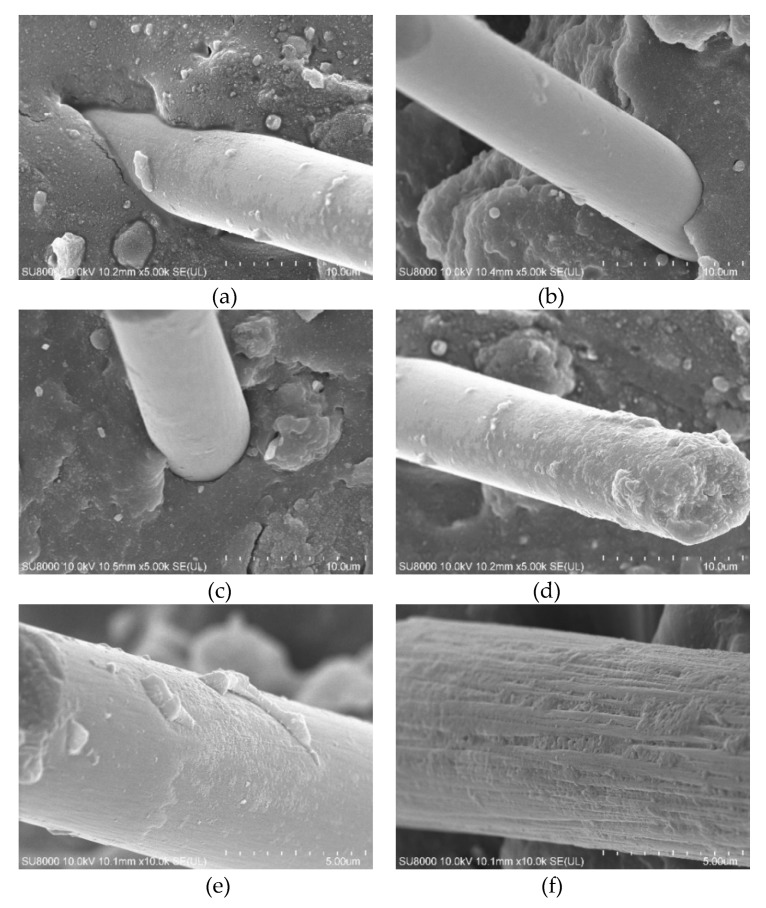
SEM picture of composites of CF modified by different concentration of dopamine. (**a**) The concentration of dopamine was 0 g/L; (**b**) the concentration of dopamine was 0.5 g/L; (**c**) the concentration of dopamine was 1 g/L; (**d**) the concentration of dopamine was 1.5 g/L; (**e**) the concentration of dopamine was 2 g/L; (**f**) the concentration of dopamine was 2.5 g/L.

The CF modified by dopamine adheres closely to the rubber matrix, and there is no difference from Figure 5. Different concentrations of dopamine solution can make the surface of the carbon fiber uneven, which makes the bonding effect between the carbon fiber and the rubber matrix different. At lower treatment concentrations (for B), a polydopamine covering could not be formed on the surface of carbon fiber completely, and stress concentration point was easily formed when the composite was stressed; with the increase in the treatment concentration, a uniform covering (for c, d, e) was formed on the surface of the carbon fiber. The interface adhesion between the carbon fiber and the rubber matrix was improved. CF was modified in the higher treatment concentration (for F), in which a non-uniform dopamine coating, which was not conducive to the adhesion between the CF and the rubber matrix, was formed.

**Figure 6:**
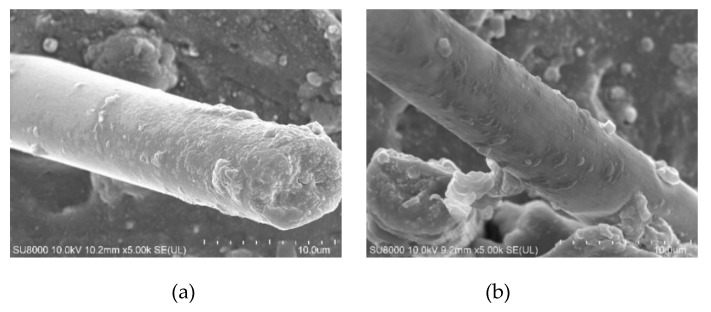

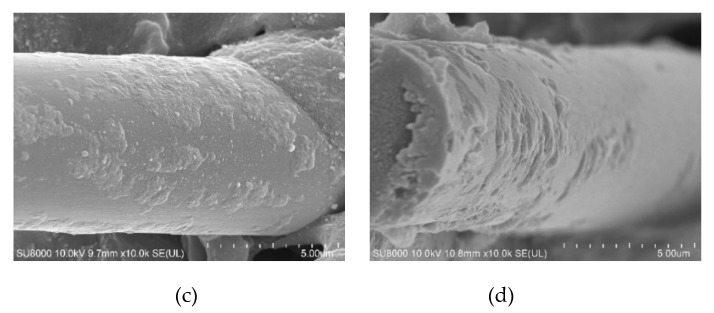
SEM picture of composites of CF modified by different processing times of dopamine. (**a**) The processing time of dopamine was 2 h; (**b**) the processing time of dopamine was 4 h; (**c**) the processing time of dopamine was 6 h; (**d**) the processing time of dopamine was 8 h;

When CF is separated from the rubber matrix, the surface morphology of NRL/CF composite prepared under different modification times is different from Figure 6. With the increase in modification time, the inhomogeneity of the dopamine coating on the surface of the carbon fiber increases, which is not conducive to the adhesion between the CF and the rubber matrix, thus reducing the adhesion performance of the composite. Dopamine coating evenly distributed, when CF was modified by dopamine for 6 h.

### 3.2. Dopamine Surface-Modified CF on the Processability of Composites

The processing properties of the NRL/CF composition were improved by CF impregnated by NFL, meaning that the value of scorching time (tc10), curing time (tc90), minimum torque (*M*_L_), maximum torque (*M*_H_), degree of crosslinking (*M*_H_ − *M*_L_) decreased, and the Mooney viscosity (ML(1 + 4)) increased from group A to B in Table 3.

The composite of NRL/CF was reinforced by dopamine-modified CF, while the values of scorching time and the degree of crosslinking increased and the curing time and Mooney viscosity decreased from groups B, C, D, E and to group F in Table 3. Meanwhile, as the curing time is shortened, the degree of crosslinking tends to rise at first, and then fall, while the Mooney viscosity tends to rise at first, then fall, then rise again with the increase in the dopamine concentration.

A layer of dopamine with a certain thickness, forming on the surface of dopamine-treated CF, reinforces the cohesion action between the CF and the rubber matrix. Thus, the non-uniform dopamine layer weakens the cohesion action, which is caused by the increasing concentrations.

The scorching time and curing time of the NRL/CF composite tend to increase due to the faintly acidic nature of dopamine, making the curing speed slow and causing the degree of crosslinking to increase slightly alongside the extension of the dopamine modification time in groups E, G, H and I in Table 3. This is mainly because the generation of a uniform dopamine layer requires a certain amount of time, which is the optimum modification point. If the dopamine modification time is insufficient, the polydopamine coating distributes unevenly on the surface of CF. However, an excessive modification time leads to thickness variations in the polydopamine layer. These non-uniform layers weaken the cohesion action.

### 3.3. Dopamine Surface-Modified CF on the Comprehensive Properties of Composite

The composite of NRL/CF has similar properties in terms of its hardness, tensile stress at 100% elongation (TS 100%), tensile stress at 300% elongation (TS 300%), tensile strength, elongation at break, resilience and DIN abrasion compared with the NR/CF composite, whereas volume resistivity is prominently improved from A to B in Table 4. Natural rubber is made from natural latex by the processes of drying and acidifying (and other procedures), in which some conductive impurities may be incorporated, which can promote its conductivity to some extent. Thus, the conductivity of the composite of NR/CF is inferior to that of NRL/CF.

The hardness of the composite of NRL/CF is basically unchanged, TS 100% and TS 300% tend to grow with the increase in dopamine concentrations from groups B, C, D, E and to group F in Table 4. When the dopamine concentrations are 1.5 g/L, the tensile strength of the composite of NRL/CF reaches the peak, which is 5.4% higher than the unmodified one (A) and the DIN abrasion decreases. This is mainly due to the even distribution of the polydopamine coating on the surface of the CF, which can reinforce the cohesion interaction between the carbon fiber and the rubber matrix, thus improving the tensile strength and the abrasion of the composite. Meanwhile, the volume resistivity of NRL/CF composites decreased first and then increased; however, overall, it is lower than that of the unmodified one (B), which correlates with the increase in the dopamine concentration. The polydopamine coating on the CF aggregated uniformly, making the interfacial interaction between the rubber matrix and the CF enhanced and the conductivity of the composite improved. At the same time, the polydopamine coating on the surface of CF will be unevenly distributed at a higher treatment concentration, meaning that the volume resistivity of the composite increases.

The hardness, resilience and abrasion of NRL/CF composites were less affected by the processing time of dopamine-modified carbon fiber. The comprehensive properties of NRL/CF composites were the best when carbon fibers were modified by the dopamine solution for 6 h. The main reason is that, before the optimal modification time is reached, the longer the time is, the larger the coverage area of the polydopamine coating on the surface of the CF is and the more uniform the thickness is, which is conducive to the interfacial adhesion between the CF and the rubber matrix. When the modification time exceeds the optimal value, the thickness of the polydopamine coating on the surface of the carbon fiber will be too thick or uneven, which will lead to a decline in the interfacial adhesion between the carbon fiber and the rubber matrix, thus reducing the mechanical properties of the rubber composite. The volume resistivity of the NRL/CF composite increases with the increase in modification time, indicating that the conductivity of the rubber sample decreases. Because of the poor conductivity of dopamine, the formation of polydopamine growing on the surface of carbon fiber will hinder the conduction pathway of the carbon fiber, thus reducing the conductivity of the NRL/CF composite.

### 3.4. Dopamine Surface-Modified CF on Thermal Conductivity of Composites

**Figure 7:**
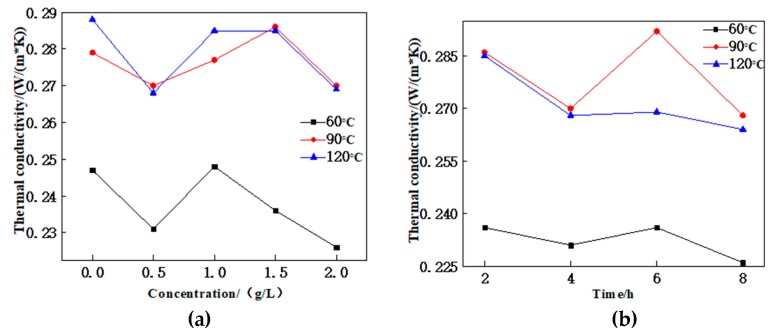
Thermal conductivity of composites: (**a**) effect of CF modified with different concentrations of dopamine on thermal conductivity of composites; (**b**) effect of processing time of dopamine-modified CF on thermal conductivity of composites.

CF has good thermal conductivity, which is dispersed evenly in the rubber matrix and overlaps to form a thermal conduction channel, enabling the NRL/CF composite to have a strong thermal conductivity. The thermal conductivity coefficient of the composite presents the tendency of rising first and then decreasing at temperatures of 60, 90 and 120 °C with the increase in the dopamine concentration from Figure 7. The main factors are that the polydopamine layer gradually changes alongside the increase in the concentration, and cannot be distributed evenly on either lower or higher modification concentrations, resulting in the formation of a clearance at the bonding point of the rubber matrix and the CF. This decelerates the conduction of heat through the composite. Thus, the dopamine layer on the surface of the carbon fiber was evenly distributed and the thermal conductivity coefficient of the composite was high at concentrations of 1 and 1.5 g/L.

The effect of CF modification time on NRL/CF composite is the same as that of modification concentrations. This is mainly because the modification time also affects the uniformity of the dopamine layer on the surface of the carbon fiber. The dopamine layer cannot be distributed evenly under either a shorter or longer modification time. When the modification time is 6 h, the dopamine layer distributes evenly on the surface of CF, and the thermal conductivity coefficient of the composite is high.

### 3.5. Dopamine Surface-Modified CF on Payne Effect of Composites

The Payne effect [21] can evaluate the interaction between composite fillers, which is usually quantified by $\Delta G^{\prime }$. The Payne effect was calculated as follows:

$$\Delta G^{\prime }=G^{\prime }(0.28\%)-G^{\prime }(40\%)$$

Generally speaking, the better the dispersion of the filler is, the lower the Payne effect is. Table 5 shows the test results of the Payne effect of the composites.

**Figure 8:**
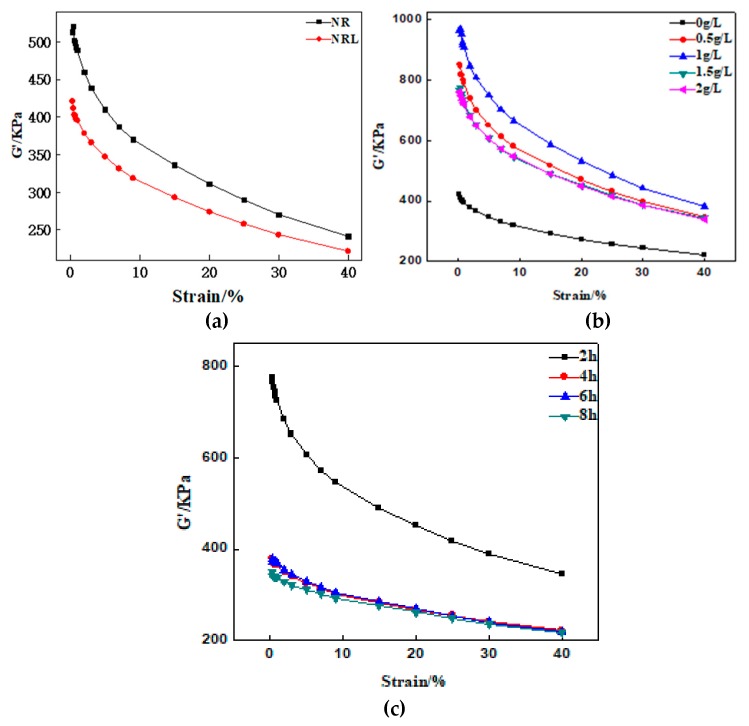
Viscoelasticity curve of composite: (**a**) viscoelasticity curve of CF with natural rubber and natural latex; (**b**) viscoelasticity curve of composites with CF modified by different concentrations of dopamine; (**c**) viscoelasticity curve of composites with CF modified by different processing times of dopamine.

The shear modulus $G^{\prime }(0.28\%)$ and $\Delta G^{\prime }$ of the NRL/CF composite is obviously lower than that of the NR/CF composite from Figure 8 and Table 5. The CF impregnated by latex can be distributed uniformly across the rubber matrix, which can weaken the interaction between the fillers, and decrease the Payne effect.

The $\Delta G^{\prime }$ of the dopamine-modified NRL/CF composite rises significantly from Figure 8, which indicates that dopamine-modified CF can restrict the rubber matrix deformation to improve the interfacial adhesion between the CF and the rubber matrix. The value of $\Delta G^{\prime }$ in terms of the dopamine-modified NRL/CF composite increases significantly from Table 5. The polydopamine layer not only reinforces the adhesion between the CF and the rubber matrix, but also causes the adhesion between CFs, so the CF aggregates and the dispersion of CF in the rubber matrix gets worse. The $\Delta G^{\prime }$ of the composite is relatively low at the dopamine concentrations of 1.5 and 2 g/L from Table 5, which indicates that the dispersion of CF in the rubber matrix and the adhesion between the CF and the rubber matrix are preferable.

The value of $\Delta G^{\prime }$ tends to decrease with the modification time, making it clear that the agglomeration of the carbon fiber is weakened and the dispersion of the carbon fiber in the rubber matrix is improved with an increase in the modification time of CF. 

### 3.6. Dopamine Surface Modification CF on Dynamic Viscoelasticity of Composites

The dynamic viscoelasticity of vulcanized rubber includes rolling resistance, wear resistance and wet-skid resistance. Rolling resistance reflects fuel economy, wear-resistance reflects the durability and service life of the tire, and the anti-skid performance is directly related to the safety of the tires, which attracts much attention.

**Figure 9:**
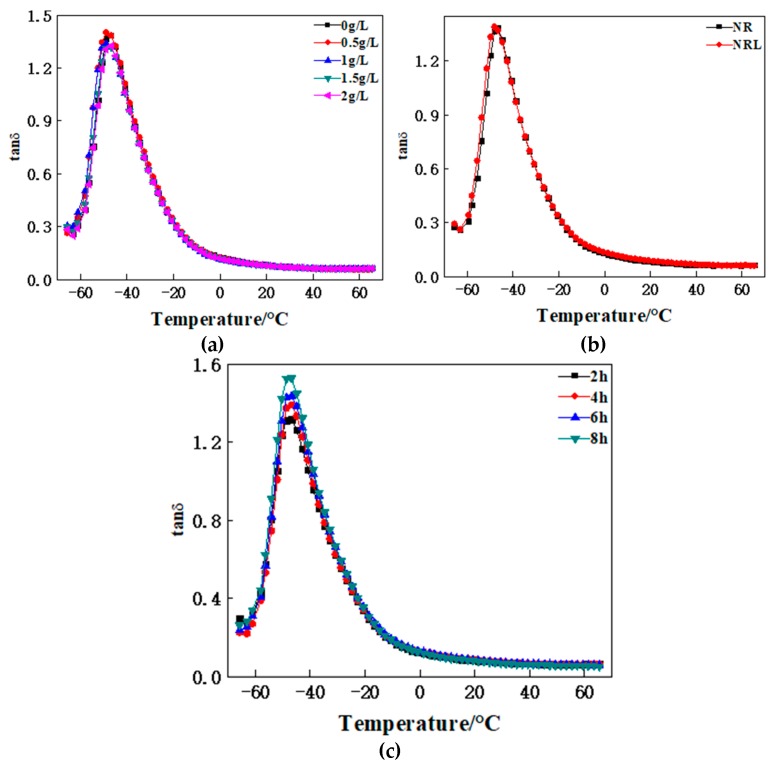
tanδ-T Curve of composites: (**a**) tanδ-T Curve of CF in natural rubber and natural latex; (**b**) tanδ-T Curve composites of CF modified by different concentration of dopamine; (**c**) tanδ-T Curve of composites of CF modified by different processing time of dopamine.

The curves of the loss factors with the temperature changes in the NRL/CF composites basically overlap with the curves of the NR/CF composites from Figure 9. This means that the properties of the composites prepared by these two methods are almost the same in terms of wet resistance and rolling resistance.

The loss factor of the composite of NRL/CF modified by dopamine of different concentrations is lower than that of the unmodified one at temperatures of 0 and 60 °C (Figure 6). The interfacial interaction between modified CF and the rubber matrix become stronger, and restrict the movement of the rubber molecule chain, making the shear modulus of the composite increase. Meanwhile, the CF becomes the stress point.

The loss factor of the composite of NRL/CF modified by dopamine of different modification times changes slightly at a temperature of 0 °C, and decreases gradually at 60 °C (Figure 6). A polydopamine layer is uniformly generated on the surface of the CF, enlarging the contact area of the CF and the rubber matrix, with the modification time increasing. The interfacial interaction is improved in order to restrict the deformation of the rubber matrix.

## 4. Conclusions

(1) Impregnated by the natural latex, the adhesion between staple carbon fiber and rubber matrix is reinforced, and the material properties of the composite are better than those prepared by the traditional method;

(2) When the concentration of dopamine was 1.5 g/L and the modification time was 6 h, the properties of the composites modified by dopamine were the best. The main reason is that when the concentration and modification time reaches an optimal value, dopamine forms a uniform polydopamine coating with a wide coverage on the surface of the carbon fiber, which significantly improves the interface adhesion between the carbon fiber and rubber matrix. 

## Figures and Tables

**Table 1:** Experimental scheme.

Sample Number	Rubber	Dopamine ^1^ Modified CF Process	
		Dopamine Concentration (g/L)	Processing Time (h)
A	NR	0	0
B	NRL	0	0
C	NRL	0.5	2
D	NRL	1	2
E	NRL	1.5	2
F	NRL	2	2
G	NRL	1.5	4
H	NRL	1.5	6
I	NRL	1.5	8

^1^ Dopamine purchased in Shanghai Aladdin Biochemical Technology Co., Ltd., Shanghai, China.

**Table 2:** Compound formulation and suppliers.

Component	Formulation (phr)	Suppliers
NR/NRL	100	Von Bundit Co. Ltd., Phuket, Thailand
CF ^1^	Variable	Toray Co., Ltd., Tokyo, Japan, Tyle: T700s, Diameter: 7 μm, Length: 3 mm
Zin Oxide	5	Hebei Shijiazhuang Zinc Oxide Factory, Shijiazhuang, China
Adhesive RA65	1.5	Wuxi Huasheng Rubber New Material Technology Co., Ltd., Wuxi, China
Carbon black N326	40	Jiangxi Black Cat Carbon Black Co., Ltd., Jiangxi, China
Stearic acid	2	Fengyi Grease Technology (Tianjin, China) Co., Ltd., China
Anti-aging agent 4020	2	Shandong shangshun Chemical Co., Ltd., Weifang, China
Resin SL3020	1	Sino Legend (China) Chemical Company Ltd., Suzhou, China
Accelerator CZ	1.5	Shandong shangshun Chemical Co., Ltd., Weifang, China
Sulfur	1.5	ChaoyangTianming Industry and Trade Co., Ltd., Beijing, China

^1^ CF is purchased from Toray Co., Ltd., Tokyo, Japan, without laboratory sizing and surface treatment.

**Table 3:** Processing properties of composite materials.

Test Item	A	B	C	D	E	F	G	H	I
tc10/min	3:54	1:58	2:33	2:29	2:32	2:27	3:29	3:23	3:46
tc90/min	8:26	7:47	6:10	5:54	5:59	5:51	8:35	7:35	8:06
*M*_L_/(dN·m)	2.04	1.95	2.01	2.46	2.48	2.32	2.40	2.57	2.43
*M*_H_/(dN·m)	17.28	16.03	16.23	16.63	16.98	16.22	16.78	17.24	17.05
*M*_H_ − *M*_L_/(dN·m)	15.24	14.08	14.22	14.23	14.50	13.90	14.38	14.67	14.26
ML(1 + 4)100 °C	44.5	47.3	47.2	46.9	47.0	47.2	48.1	48.0	47.7

**Table 4:** The physical and mechanical properties of composites.

Test Item	A	B	C	D	E	F	G	H	I
Hardness/°	64	65	65	66	66	66	66	66	67
TS 100% ^1^/MPa	3.21	2.97	3.01	2.93	3.15	2.97	2.64	2.68	2.35
TS 300% ^2^ /MPa	12.80	12.5	12.38	12.47	12.72	12.43	13.23	13.81	11.90
TS ^3^/MPa	24.78	24.8	24.42	25.75	26.15	25.44	26.42	27.45	26.13
Elongation at break/%	510.36	490.04	552.96	519.68	544.44	550.52	490.84	509.91	524.16
Resilience/%	71.98	71.84	70.87	71.24	71.98	71.46	72.03	71.99	72.14
Abrasion/cm^−3^	0.139	0.140	0.151	0.148	0.140	0.142	0.141	0.139	0.140
Volume resistivity/Ω·cm	1.04 × 10^6^	4.71 × 10^7^	2.52 × 10^7^	2.62 × 10^6^	1.02 × 10^7^	1.61 × 10^7^	1.01 × 10^7^	1.54 × 10^7^	2.8 × 10^8^

^1^ Tensile stress at 100% elongation (TS 100%); ^2^ tensile stress at 300% elongation (TS 300%); ^3^ tensile strength (TS).

**Table 5:** The test results of Payne effect of composites.

	A	B	C	D	E	F	G	H	I
$G^{\prime }(0.28\%)$/KPa	513.25	421.13	851.28	963.64	765.4	759.94	396.5	390.44	385.63
$G^{\prime }(40\%)$/KPa	241.47	221.9	346.54	382.96	344.6	338.82	238.47	229.98	252.97
$\Delta G^{\prime }$/KPa	271.78	199.23	504.74	580.68	420.8	421.12	158.03	160.46	132.7
